# Book Reviews

**Published:** 1894-09

**Authors:** 


### *BOOK REVIEWS.*

A SYSTEM OF GENITO-URINARY DISEASES, SYPHILOLOGY AND DERMATOLOGY. By Various Authors. Edited by Prince A. Morrow, A.M. M.D., Clinical Professor of Genito-Urinary Diseases, etc., etc., Surgeon to Charity Hospital, etc. With Illustrations. In three Volumes. Volume III. DERMATOLOGY. New York, D. Appleton & Co., 1894.

This exhaustive work on Dermatology is a collection of articles upon the various skin diseases, written for the most part by authors especially fitted to handle the subjects assigned them.

Beginning with the anatomy and physiology of the skin, then semeiology, etiology, and classification, the various dermatoses are taken up in order of classification.

The classification is as follows: Inflammations, Hemorrhages, Hypertrophies, Atrophies, New Growths, Neuroses, Diseases of the Appendages of the Skin, and Parasitic Diseases. In this classification Morrow has followed, "with certain changes," Crocker's modification of Hebra's system, adopting the plan of grouping those diseases together which are most closely related pathologically.

Many conditions are now recognized as distinct which were formerly classed in a general group.

The illustrations show a marked improvement upon former methods of showing the characters of skin diseases in pictorial form.

Lichen ruber and pityriasis pilaris are synonymously used, lichen planus and lichen scrofulosus completing the true lichen group according to this work. In Europe, especially, this lichen question is still chaotic, lichen ruber and lichen planus being by many considered identical, by others distinct, while pityriasis rubra pilaris (Devergie) is considered a separate disease.

But there are many views of lichen and many lichens, the views and the number of distinct lichens being almost as numerous as the observers.

Although admittedly parasitic, the tuberculoses of the skin are described under "New Growths." Tuberculosis cutis, lupus vulgaris, tuberculosis verrucosa and scrophuloderma appear here. The "psorosperm" question is not decided, being left as yet in an uncertain state.

The book is really a collation of the views of leading authorities upon the various skin diseases, the verdict of the writer of each article being in favor of what appears as positively proven.

As a reference the volume is perhaps too full, as a text-book perhaps too complete, as an up-to-date exponent of dermatology it is unequalled. M. B. H.

---

MACROBIOTIC, or Our Diseases and Our Remedies. For Practical Physicians and People of Culture. By Julius Hensel, Physiological Chemist. Boericke & Tafel, 1011 Arch Street, Philadelphia.

Herein is wisdom in large and solid chunks, which the author was constrained to lay before "practical physicians and people of culture" by the "consideration that I was in advance of the majority of medical practitioners, from having commenced the study of medicine in later life, after having been an apothecary for twenty years."

Illustrative of this physiologico-chemico-medical erudition are: "I herein ascribe the origin of internal diseases to a diminished *electric force*." "Diphtheria, or children's catarrh, begins with a stagnation of blood in the thymus gland." "Napoleon suffered from epilepsy and the itch. The cause of both was an insufficiency of earthy substances in his diet. Through a deficiency of sulphur part of the mucous membranes become changed into itch-worms," etc. "Physicians have heretofore been ignorant of the fact that the spleen acts as a source of electricity. I myself have solved the question," etc. The graphic formulæ on the title page look like crochet designs. To one of these the author attaches *Cum Deo*, but we fail to catch the atomic connection. Here is at last a solution of the great problem of *Macrobiosis*, or long life. *And there shall be no more death. Eureka! Cum Deo!!*

---

PAIN. In its Neuro-Pathological, Diagnostic, Medico-Legal and Therapeutic Relations. By J. L. Corning, M.D., Consultant in Nervous Diseases to St. Francis Hospital; Fellow of the New York Academy of Medicine; Author of "A Treatise on Headache and Neuralgia, etc." Illustrated. Price, $1.75. J. B. Lippincott Co., Philadelphia.

The author of this book on Pain in all its relations discusses his subject with that fullness and familiarity which characterize all his works on the phenomena of nervous disease. The expression, "physiology of pain," is objectionable. Pain is not physiological. The chapter on the Pathology of pain discusses neuritis in full.

Pains located in definite nerve areas, neuralgia, pains associated with the rheumatic and gouty diathesis are also described. A chapter is devoted to the medico-legal aspects of pain as a subjective symptom of spinal concussion. The second part relates to the Special Therapeutics of Pain. The author here describes in detail the importance and application of the "rest treatment" for the troublesome nervous symptoms following prolonged pain. He also tries to define the proper status of opium, the bromides and the coal-tar preparations, with the suitable indications for each. Electrolysis and hypnotism are also discussed. We think any one will find this book of great service in the management of one of our most troublesome symptoms.

---

A TREATISE ON HEADACHE AND NEURALGIA. By J. Leonard Corning, M.A., M.D., Consultant in Nervous Diseases to St. Francis Hospital, Fellow of New York Academy of Medicine, etc. Third edition. Price, $2.75. E. B. Treat, 5 Cooper Union, New York.

This is only a revision of the author's previous treatise on this subject, which has been so favorably received by the profession. In this volume there is an excellent and exhaustive appendix by Dr. David Webster on "Eye-strains as a Cause of Headache." A chapter also on "Localization of the Action of Remedies on the Brain" is included. Dr. Corning has made a thorough presentation of the phonomena and treatment of the troublesome conditions, and his book is deserving of the highest confidence.

---

SYLLABUS OF THE OBSTETRICAL LECTURES IN THE MEDICAL DEPARTMENT OF THE UNIVERSITY OF PENNSYLVANIA. By Richard C. Norris, A.M., M.D., Demonstrator of Obstetrics, University of Pennsylvania. Third Edition. W. B. Saunders, Philadelphia.

This valuable book contains a series of lectures which cover the field of Physiology and Pathology of Gestation and Labor, also the Diseases of the Fetus in Utero, with the causes and latest methods of treatment of the puerperal diseases. J. L. C.

